# Acute appendicitis: transcript profiling of blood identifies promising biomarkers and potential underlying processes

**DOI:** 10.1186/s12920-016-0200-y

**Published:** 2016-07-15

**Authors:** Lakhmir S. Chawla, Ian Toma, Danielle Davison, Khashayar Vaziri, Juliet Lee, Raymond Lucas, Michael G. Seneff, Aoibhinn Nyhan, Timothy A. McCaffrey

**Affiliations:** Department of Anesthesiology and Critical Care Medicine, The George Washington University Medical Center, 2300 I Street, NW Ross 443, Washington, DC, 20037 USA; Department of Medicine, Division of Genomic Medicine, The George Washington University Medical Center, 2300 I Street, NW Ross 443, Washington, DC, 20037 USA; Department of Surgery, The George Washington University Medical Center, 2300 I Street, NW Ross 443, Washington, DC, 20037 USA; Department of Microbiology, Immunology, and Tropical Medicine, The George Washington University Medical Center, 2300 I Street, NW Ross 443, Washington, DC, 20037 USA; Department of Emergency Medicine, The George Washington University Medical School and GW Medical Faculty Associates, Washington, DC, USA; The Department of Medicine, Veterans Affairs Medical Center, The George Washington University Medical Center, 2300 I Street, NW Ross 443, Washington, DC, 20037 USA

**Keywords:** Appendicitis, Transcript profiling, Biomarkers, Interleukin-8 receptor, Alkaline phosphatase, Defensin

## Abstract

**Background:**

The diagnosis of acute appendicitis can be surprisingly difficult without computed tomography, which carries significant radiation exposure. Circulating blood cells may carry informative changes in their RNA expression profile that would signal internal infection or inflammation of the appendix.

**Methods:**

Genome-wide expression profiling was applied to whole blood RNA of acute appendicitis patients versus patients with other abdominal disorders, in order to identify biomarkers of appendicitis. From a large cohort of emergency patients, a discovery set of patients with surgically confirmed appendicitis, or abdominal pain from other causes, was identified. RNA from whole blood was profiled by microarrays, and RNA levels were filtered by a combined fold-change (>2) and *p* value (<0.05). A separate set of patients, including patients with respiratory infections, was used to validate a partial least squares discriminant (PLSD) prediction model.

**Results:**

Transcript profiling identified 37 differentially expressed genes (DEG) in appendicitis versus abdominal pain patients. The DEG list contained 3 major ontologies: infection-related, inflammation-related, and ribosomal processing. Appendicitis patients had lower level of neutrophil defensin mRNA (DEFA1,3), but higher levels of alkaline phosphatase (ALPL) and interleukin-8 receptor-ß (CXCR2/IL8RB), which was confirmed in a larger cohort of 60 patients using droplet digital PCR (ddPCR).

**Conclusions:**

Patients with acute appendicitis have detectable changes in the mRNA expression levels of factors related to neutrophil innate defense systems. The low defensin mRNA levels suggest that appendicitis patient’s immune cells are not directly activated by pathogens, but are primed by diffusible factors in the microenvironment of the infection. The detected biomarkers are consistent with prior evidence that biofilm-forming bacteria in the appendix may be an important factor in appendicitis.

**Electronic supplementary material:**

The online version of this article (doi:10.1186/s12920-016-0200-y) contains supplementary material, which is available to authorized users.

## Background

Abdominal pain is a major cause of hospital visits, accounting for about 10 % of 62 million visits per year by adults who present at an emergency department (ED) for non-injury causes [1]. Acute appendicitis is one of the most common causes of abdominal pain and results in nearly 750,000 ED visits with approximately 250,000 appendectomies performed annually. Globally, a small but significant portion of the operations are “negative appendectomies”, resulting in the removal of a non-inflamed appendix due to misdiagnosis [2–4], reported as high as 17-28 % outside the United States (US) and Western Europe [5, 6].

Prior to the widespread availability of computed tomography (CT) scans, the accurate diagnosis of appendicitis could be challenging, and in places where CT is still not available, the Alvarado score of clinical characteristics is a widely used diagnostic tool [5, 6]. Currently in the US, CT scanning is the ‘gold standard’ for the diagnosis of appendicitis, with magnetic resonance imaging (MRI) being a reasonable alternative in pregnant women [7], and ultrasound sonography being an acceptable alternative for preliminary diagnostics to avoid radiation [8]. While CT is the most sensitive and specific diagnostic tool for appendicitis [9, 10], and used in almost 98 % of patients undergoing appendectomy in the US [11], CT scanning carries a significant radiation exposure, and epidemiologic data suggest that radiation exposure can increase the risk of developing a future malignancy [12]. This issue is of particular concern in children because they are more sensitive to the hazards of radiation, they are among the most common patients to present to the ED with abdominal pain, and have the highest rate of misdiagnosis [10, 13]. In an attempt to reduce the damaging effect of CT scans, several clinical trials are examining the diagnostic utility of lower doses of radiation, primarily in children [14–16].

In order to understand the landscape of the immune reaction to appendicitis, we tested the hypothesis that microarray profiling of whole blood RNA would identify blood biomarkers of appendicitis. Toward that goal, we employed genome-wide profiling of RNA transcripts in whole blood RNA of patients presenting at the ED for abdominal pain, resulting in confirmed appendicitis versus other abdominal abnormalities. Ultimately, there is the potential to use blood RNA biomarkers as a prescreening method to utilize CT scanning more appropriately, and to improve diagnosis in areas where CT scans are unavailable.

## Methods

### Subjects

#### Ethics statement

The protocol of this observational study was approved by the Institutional Review Board of The George Washington University, and all subjects gave informed consent. From a cohort of 270 patients presenting to the ED for various reasons, a subset of 40 subjects with a principal complaint of abdominal pain, and who met inclusion/exclusion criteria (Additional file 1: Table S1), were identified, and divided into a discovery set of 20 patients, and a validation set of 20 patients for transcript profiling of whole blood RNA by microarray.

##### Discovery Set

For the discovery set, we employed 20 subjects who presented to the ED who were undergoing CT scanning. In order to meet criteria, the patient undergoing the CT scan must have had appendicitis suspected in the differential diagnosis. *Appendicitis Patients*: Patients with appendicitis were diagnosed by CT scanning (*n* = 11), and had research blood samples drawn by venipuncture after anesthetic induction, but prior to skin incision for appendectomy. All cases of appendicitis were confirmed by surgical inspection and histopathology, including mucosal ulceration and neutrophil infiltration of the lamina propria and/or perivascular space. *Control Patients*: Patients included in the control arm (*n* = 9) were patients who were found not to have appendicitis, by both CT scanning and clinical follow-up. This included patients with reported abdominal pain (ABD), later found to be caused by diverticulitis, colitis, or other gastrointestinal pathologies, but not clinically associated with appendicitis. Blood was drawn at study enrollment for these patients.

##### Validation Set: control patients

Because appendicitis can involve infection, 5 patients with lower respiratory tract infections (LRI) in the ED were enrolled as an ‘infection’ control. Also, as a control for surgical factors, 5 patients undergoing elective ventral hernia or inguinal hernia repair (HER) were enrolled, and they were compared with 9 new patients with surgically confirmed appendicitis (APP). In all surgical patients, including appendicitis and hernia repairs, research blood samples were drawn by venipuncture after anesthetic induction, and prior to skin incision. Two patients, (1 HER, 1 APP) were excluded due to technical complications in RNA purification or microarray analysis. Clinical diagnoses on all patients analyzed are available in Additional file 2: Table S2.

### Blood samples

Blood was drawn in 3.2 % sodium citrate tubes for frozen plasma samples, in Tempus Blood RNA tubes (ABI) for genome-wide RNA profiling, and in BD Vacutainer K2 tubes for complete blood counts with differentials.

### RNA purification for transcript profiling

Tempus Blood RNA preservation tubes were stored at −80 ° C and then thawed at 37 ° C prior to processing according to manufacturer’s methods. Total RNA was purified from whole blood using Tempus Blood RNA kit (ABI), followed by an aggressive DNAse treatment. Briefly, the preserved whole blood was pelleted at 3000 x g for 30 min in a 4 °C refrigerated centrifuge, redissolved in lysis buffer, and nucleic acids were bound to a column. After washing, nucleic acids were eluted with nuclease-free water and quantified with a NanoDrop ND-1000 spectrophotometer. DNA was eliminated by aggressive DNAse treatment (TurboDNAse, Ambion) at 2 U/10 μg nucleic acids, followed by affinity removal of the DNAse. The remaining RNA was quantified by 260/280 ratio by NanoDrop, and RNA integrity was evaluated by capillary electrophoresis on a Bioanalyzer 2100 (Agilent). RIN scores >7 were considered acceptable for further sample processing and did not differ between groups.

### Microarray expression profiling and analysis

Purified RNA (100 ng) was labeled with the Illumina cRNA synthesis kit and hybridized to Illumina Human HT-12v4 Expression BeadChip arrays containing 45,966 probes derived from the NCBI RefSeq release 38. The arrays were washed and then fluorescence was quantitated on an Illumina HiScan.

The fluorescence levels per bead were converted to transcript levels using Illumina GeneStudio, which averaged ~30 beads per transcript to produce a mean expression level for each of the 46 K transcripts. Raw BeadChip fluorescence values were imported into GeneSpring GX12.5 with normalization to the 75^−^percentile of expression, but without baseline transformation. The main effect of identifying differentially expressed genes (DEG) with respect to appendicitis versus controls was achieved by a combined filter for a *p* value <0.05 on *t* test without correction for multiple testing, and 2) fold change > 2.0. The DEG list was further analyzed for gene ontologies using DAVID [17]. Using the DEG list, a partial least squares discriminant (PLSD) prediction model was built in GeneSpring and internally validated with a Leave One Out Cross Validation (LOOCV) algorithm. The PLSD model was externally tested by applying the algorithm to a separate validation set of microarray samples not involved in building the model.

### Validation by droplet digital PCR (ddPCR)

In addition to the patient samples used for the discovery and validation microarray studies, an additional 29 patients (12 APP, 17 ABD) were drawn from the same cohort, to compose a cohort for ddPCR validation (30 APP, 25 ABD, 5 HER). RNA was purified from Tempus-preserved whole blood as described above and DNAse-treated prior to quantitative PCR using a ddPCR system (BioRad QX200). Total RNA was reverse transcribed with an RNAseH+ reverse transcriptase (iScript, BioRad) using random hexamer primers. The cDNA was purified and amplified with transcript-specific primers for ALPL, IL8RB, DEFA1, and ACTB (Additional file 3: Table S3). ddPCR uses clear oil to create thousands of individual nanoliter-sized droplets containing cDNA and PCR reagents with a fluorescent detection dye EVAgreen. The number of positive droplets is proportional to the abundance of the cDNA target of interest, and can be calculated in absolute quantity from a Poisson distribution. To account for variations between RNA preparations, the quantity of target transcripts ALPL, IL8RB, and DEFA1 was expressed as a % of the ACTB levels in the sample.

### Statistical analysis

Summary statistics are reported as mean and standard error of measurement (SEM). Comparisons between groups are made by an unpaired Student’s t test with correction for multiple testing using the method of Benjamini and Hochberg, unless otherwise specified. Correlations between multiple measures of samples were computed as the Pearson r statistic.

## Results

### Clinical parameters

As shown in Table 1, the clinical parameters between patients presenting with appendicitis versus other abdominal indications in the discovery set were generally similar. Age, gender, and body mass index (BMI) were comparable, although the appendicitis patients in the discovery set were all Caucasian. Notably, white blood cell (WBC) counts were comparable, but appendicitis patients had a slightly higher percentage of neutrophils, which was not statistically significant (77.18 % vs 70 %, NS). Appendicitis patients had significantly lower blood creatinine level (0.78 vs 1.54 mg/dL, *p* = 0.03 uncorrected). The two groups did not yield significantly different RNA quantities from blood, and the efficiency of RNA amplification for microarray labeling was similar.

**Table 1:** Demographic, clinical, and laboratory findings of patients in the discovery cohort

		Unit	APP (11)	ABD (9)
Gender		%M	54.55	55.56
Age	Mean	Years	40.73	45.89
	SD		15.45	15.54
BMI	Mean	BMI	24.51	26.44
	SD		4.92	4.48
Race		%C	100.00	55.56
		%AA	0.00	44.44
Smoker		%	18.18	11.11
Duration of Symptom	Mean	Hours	29.45	32.75
	SD		18.68	30.65
Temperature	Mean	Celsius	36.97	36.80
	SD		0.47	0.38
WBC	Mean	K/ul	13.06	13.23
	SD		6.44	30.65
Elevated Neutrophils	>75 %	%	55.00	37.50
Neutrophils	Mean	%WBC	77.18	70.00
	SD		8.76	10.14
Creatinine	Mean		0.78	1.54*
	SD		0.13	1.06
pH	<7.35	%	0.00	11.11
Na < 130		%	0.00	0.00
HCT < 30		%	0.00	11.11
Glu > 250		%	0	0
Total RNA conc.	Mean	ng/ul	102.36	66.48
Immunosupressed	SD		72.49	34.06
Folds amp.	Mean	fold	67.96	64.13
Antibiotic use	SD		60.48	35.81
Defensin Score	Mean	RNA level	1.26	2.62*
	SD		0.92	1.46

^*^indicates *p* < 0.05 (uncorrected t-test probability)

% indicates the percent of patients exhibiting that trait, unless otherwise indicated

### Identification of RNA biomarkers for appendicitis in whole blood

**Fig. 1:**
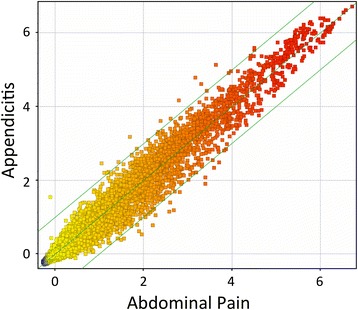
Scatterplot of transcript levels in patients with appendicitis. Whole blood RNA from patients with acute, surgically confirmed appendicitis (*n* = 11) or abdominal pain (*n* = 9) was profiled for the expression level of 45,966 transcripts on Illumina BeadChip Arrays (12v4). The expression level of each transcript was averaged within groups and plotted on a log2 scale to reveal transcripts which differ between more than 2-fold between groups (outside parallel lines)

**Fig. 2:**
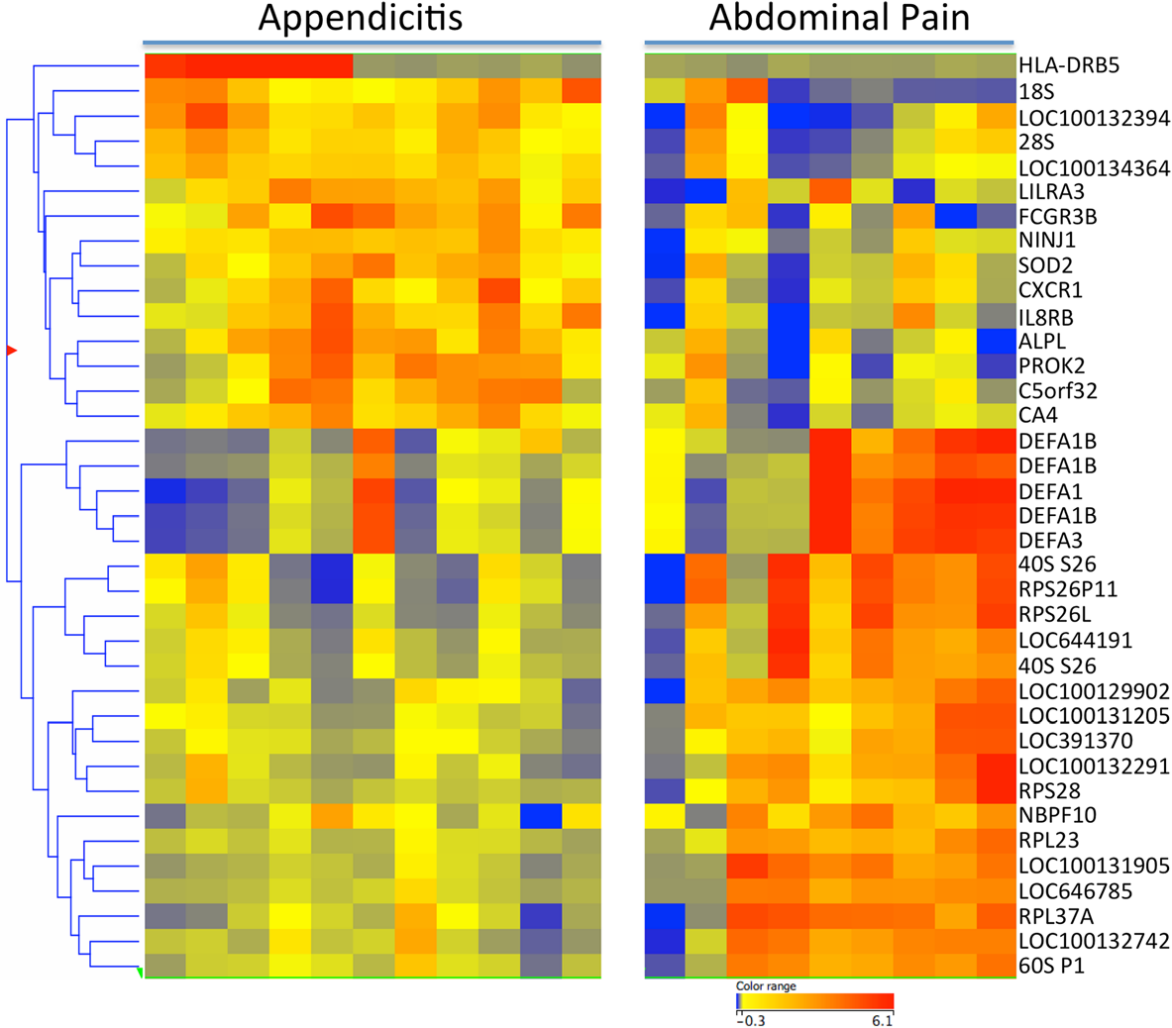
Hierarchical clustering of 37 differentially expressed genes in appendicitis patients. Transcripts which differed between groups by >2-fold with a t-test probability of <0.05 (uncorrected) were identified by combined filtering. Following a per-gene normalization, DEGs were subjected to hierarchical clustering to identify patterns of covariance among the transcripts. The upper block of transcripts from HLA-DRB5 to CA4 are relatively higher in APP patients (red) compared to patients with other types of abdominal pain (yellow to blue). Conversely, transcripts from defensins (DEFA) and ribosomal transcripts, were relatively lower in APP than abdominal pain patients

A scatterplot of the expression patterns in the 2 groups (Fig. 1) suggested that there was excellent linearity of quantitation over roughly 7 log2 orders of magnitude, with globins being the most highly and identically expressed transcripts between groups. By comparing the expression profiles of the two groups, and filtering for both a *t*-test probability <0.05 and a fold-change of >2.0, 37 transcripts were identified as significantly differentially expressed (Table 2). Hierarchical clustering of the 37 DEG was conducted to observe the pattern of covariance of the transcripts in these patients. A heatmap of the expression of these 37 transcripts across all 20 patients in the discovery set is shown in Fig. 2.

**Table 2:** Differentially expressed genes (DEG) sorted by functional grouping

Probe		Fold		Expression Level			
ID	p Val	Change		ABDOM	APPDX	Definition	Symbol
Chemokines and immune-related							
3440669	0.008	2.02	⬆	1.85	2.86	Chemokine C-X-C receptor 1	CXCR1
2900327	0.003	2.59	⬆	2.80	4.17	Interleukin 8 receptor, ß (CXCR2)	CXCR2
1450139	0.004	3.07	⬆	3.17	4.79	Fc frag of IgG receptor IIIb (CD16b)	FCGR3B
6370315	0.017	3.16	⬆	−0.11	1.55	MHC class II, DR beta 5	HLA-DRB5
6110037	0.007	2.36	⬆	2.38	3.62	Leukocyte IgG-like receptor A3	LILRA3
Defensins							
4540239	0.019	2.80	⬇	3.39	1.91	Defensin, alpha 1	DEFA1
870477	0.024	2.29	⬇	2.60	1.40	Defensin, alpha 1B (3 probesets)	DEFA1B
2970747	0.017	2.69	⬇	2.58	1.15	Defensin, alpha 3, neutrophil-spec.	DEFA3
Translation and protein synthesis							
3180609	0.002	2.69	⬆	1.04	2.47	18S ribosomal RNA, non-coding	18S rRNA
6280504	0.005	2.05	⬆	1.20	2.23	28S ribosomal RNA, non-coding	28S rRNA
3190348	0.007	2.01	⬇	2.16	1.15	60S acidic ribosomal protein P1	RPLP1
6270307	0.006	2.04	⬇	2.04	1.01	40S ribosomal protein S26 (3 sets)	RPS26
380575	0.000	2.14	⬇	1.49	0.39	Ribosomal protein L23	RPL23
990273	0.012	2.48	⬇	3.39	2.08	Ribosomal protein L37a	RPL37A
650349	0.008	2.00	⬇	2.20	1.19	Ribosomal protein S28	RPS28
Stress and injury related							
6100356	0.002	2.84	⬆	3.63	5.14	Alkaline phosphatase, liver/bone	ALPL
6380672	0.001	2.11	⬆	1.42	2.50	Carbonic anhydrase IV	CA4
1510681	0.012	2.01	⬇	3.56	2.55	Neuroblastoma breakpt family 10	NBPF10
7380706	0.001	2.10	⬆	2.61	3.68	Ninjurin 1	NINJ1
1030463	0.004	2.49	⬆	3.30	4.62	Prokineticin 2	PROK2
3890326	0.011	2.02	⬆	3.43	4.44	Superoxide dismutase 2, mitochon.	SOD2
Minimally annotated							NCBI
6420563	0.023	2.00	⬇	3.85	2.85	LOC100129902	RPS29P11
650735	0.001	2.09	⬇	1.86	0.79	LOC100131205	RPL21P28
6650603	0.000	2.66	⬇	1.95	0.54	LOC100131905	RPS27P21
7150414	0.003	2.31	⬇	2.26	1.06	LOC100132291	RPS27P29
4670634	0.003	2.81	⬆	1.69	3.18	LOC100132394	retired
6580017	0.009	2.18	⬇	2.81	1.69	LOC100132742	RPL17L
2630347	0.001	2.04	⬆	1.17	2.21	LOC100134364	retired
3390674	0.002	2.01	⬇	2.11	1.10	LOC391370	RPS12P4
1170551	0.001	2.19	⬇	1.55	0.42	LOC646785	RPS10P13
6960373	0.013	2.00	⬇	2.23	1.23	LOC644191	RPS26P8
4540241	0.005	2.15	⬆	1.10	2.21	C5orf32	CYSTM1

### Functional analysis of DEG transcripts

Of the well annotated transcripts, several had prior published relationships to infection, immunity, or inflammation, or stress/injury: notably, alkaline phosphatase liver/bone/kidney isoform (ALPL), carbonic anhydrase IV (CA4), chemokine (C-X-C motif) receptor 1 (CXCR1/IL-8 receptor α), defensin α1 (DEFA1), defensin α3 (DEFA3), IgG Fc receptor IIb (FCGR3B/CD16B), interleukin 8 receptor ß (CXCR2/IL8RB), ninjurin 1, (NINJ1), prokinectin 2 (PROK2), and superoxide dismutase 2 (SOD2). In addition to their logical connection to appendicitis, which often has an infectious etiology, certain aspects of this expression pattern increase the confidence that some of these changes are non-random: 1) multiple probe sets identifying the same transcript (DEFA1), 2) ‘hits’ on highly related transcripts, such as DEFA1 and DEFA3, as well as CXCR1 (IL8 receptor α) and CXCR2 (IL8 receptor ß).

#### Defensins

To understand the defensin pathway, the 5 α-defensin transcripts in the DEG list, which are all variant transcripts from the DEFA locus at 8p21.3, were averaged to create a ‘defensin score’, and then compared between groups (Table 1). Using a threshold determined by the mean of all 20 patients (1.87), 6 of 9 (67 %) patients with other abdominal disorders showed elevated defensins, while only 1 of 11 (9 %) of appendicitis patients had elevated defensin mRNA (see defensin cluster in Fig. 2). Surprisingly, the defensin score was essentially uncorrelated with white blood cell count (WBC) (*r* = 0.07) and neutrophil % (*r* = 0.15).

#### Other immune/inflammatory pathways

Interestingly, 3 of the 37 DEG (LILRA3, CXCR1/IL8RA, FCGR3A), which were higher in appendicitis patients compared to abdominal pain patients, are near or exact matches to transcripts discovered previously as down-regulated by exposure of isolated human neutrophils to *E. Coli* [18]. However, across the 20 patients, they were not inversely correlated with defensin expression (LILRA = 0.02, CXCR1 = −0.02, FCGR3A = −0.33), suggesting they are regulated independently of infectious markers.

#### Ribosomal transcripts

While it is widely assumed that ribosomal RNAs (rRNA), such as 18S and 28S non-coding RNAs are ‘invariant’, or ‘housekeeping’ transcripts, there is considerable evidence that they are carefully regulated in cases such as granulocyte activation [19], and differ significantly in prostate cancer [20], and in hepatitis C infected livers [21]. In fact, early studies with PHA-activated human lymphocytes demonstrated as much as 8-fold increases in rRNA levels within 20 h [22, 23]. Furthermore, if the observed changes were due to some type of loading or processing anomaly, then we would expect all of the ribosomal RNAs to be affected in the same direction, when in fact, 18S and 28S noncoding transcripts were increased in appendicitis, but most of the transcripts coding for ribosomal proteins were decreased, suggesting that this is a regulated process.

#### Minimally annotated transcripts

Of the 37 DEG, 11 transcripts were minimally annotated, i.e. ‘predicted transcript’, but further manual annotation using NCBI Gene revealed high likelihood assignments. Remarkably, 8 of the 11 transcripts were identified as ribosomal protein pseudogenes, which is quite unlikely to have occurred by chance. Two transcripts have been discontinued, and the eleventh was identified as CYSTM1 (C5ORF32), which is a cysteine-rich transmembrane module-containing protein that 2-hybrid screens identified as an inhibitor of the glucagon-like peptide 1 receptor (GLP-1R) [24].

### Prediction of appendicitis from DEG

**Fig. 3:**
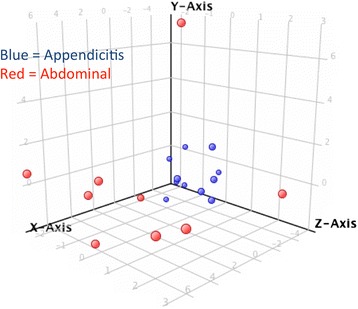
Partial Least Squares Discriminant (PLSD) Model for classification of appendicitis from RNA biomarkers. DEGs were analyzed by PLSD to compose a classification model for appendicitis based on RNA biomarkers in blood. The 3D plot shows the 20 patients in the discovery set as partitioned by the first 3 of 4 latent factors in the PLSD model. The red spheres represent abdominal pain patients (*n* = 9), and blue shows the cluster of appendicitis patients (*n* = 11), as a function of the t0 latent factor (X-axis), the t1 factor (Y-axis), and the t2 factor (Z-axis). The majority of patients (7/9) are accurately classified by the t0 component alone

The PLSD model built on the 37 DEG list, was 100 % accurate and specific within the discovery set, which is not surprising given the ability of PLSD models to accurately ‘fit’ data to outcomes. As shown in Fig. 3, the first 3 latent factors in the PLSD model demonstrate tight clustering of the appendicitis patients (blue) distinct from patients presenting with other abdominal pain (red). Clearly, 7 of 9 abdominal patients can be discriminated by only the first latent factor (t0, X-axis). Two abdominal patients, one with a GI bleed and one with diverticulitis, are poorly discriminated by the t0 latent factor shown in the X-axis, but are readily discriminated by one of the two other factors (Y or Z axis). To determine whether all 37 transcripts were necessary for prediction, 16 transcripts with a loading of >0.2 in the PLSD model were used to rebuild a new PLSD prediction model (Additional file 4: Table S4). This smaller model, which omitted the defensins, remained quite strong, predicting 100 % of abdominal cases, 90.9 % of appendicitis cases, for an overall accuracy of 95 %.

### Validation of PLSD prediction model in unrelated samples

To determine the robustness of the prediction model, a separate group of patients derived from the same overall cohort were similarly processed for whole blood RNA, and hybridized independently to Illumina HT 12v4 Beadchip arrays. With only minimal normalization to correct for minor loading and hybridization differences, the PLSD prediction model was applied to the normalized values for the 37 transcripts in the model. The PLSD prediction model correctly identified 8 of 9 true appendicitis patients (88.9 %) and predicted 3 of 4 patients (75 %) with hernias as being ‘abdominal pain’. Nearly 90 % sensitivity in an unrelated cohort quantified on a different microarray run is encouraging toward the potential robustness of the model. Notably, the PLSD model includes no clinical variables, such as fever or white cell count.

### Behavior of the RNA biomarkers in non-appendicitis infections

**Fig. 4:**
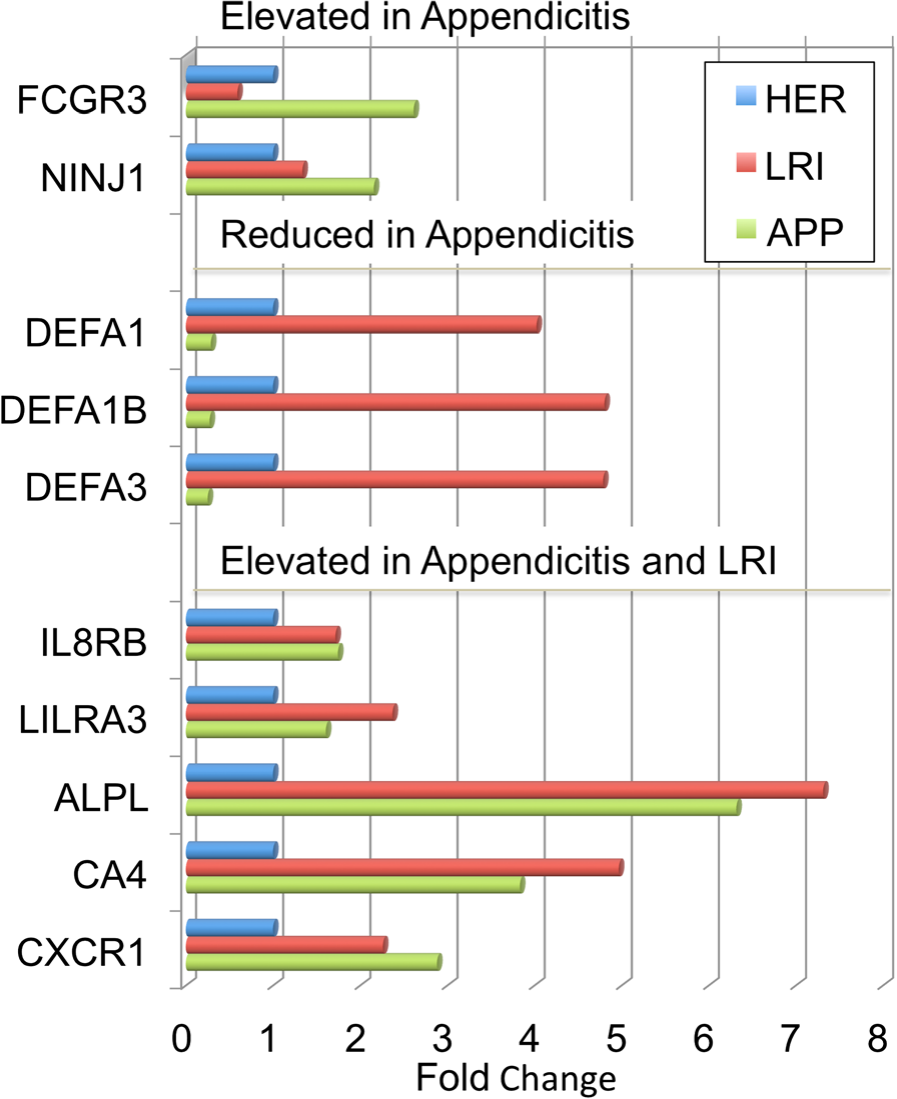
Behavior of DEG biomarkers in a validation cohort. The 37 DEG biomarker set was applied to transcript expression levels in patients being treated for either appendicitis (APP, green bars), lower respiratory infection (LRI, red bars), or hernias (HER, blue bars). Representative transcripts, such as Fc gamma receptor 3 (FCGR3) and ninjurin 1 (NINJ1) are shown, in which the transcript behaves with relatively selective induction in APP, relative to HER or LRI. Conversely, transcripts in the defensin family (DEFA1, DEFA3), are significantly elevated in HER patients, relative to APP, but are strikingly induced in LRI patients. Most transcripts, such as alkaline phosphatase (ALPL) and the CXCL8 (IL-8) receptors (CXCR2/IL8RB, CXCR1), were induced in both APP and LRI patients

In 5 patients clinically diagnosed with LRI, which were not included in PLSD training, the model predicts 4 of 5 as appendicitis (80 %), suggesting that the model may be sensitive to generalized infectious or inflammatory signals in blood. Using the 16 DEG model, only 60 % were diagnosed as appendicitis. As shown in Fig. 4, some transcripts, such as FCGR3 and NINJ1, were relatively selectively elevated in APP, but not LRI. Other transcripts, especially defensins, were much more sensitive to LRI than APP, showing 4–5 fold elevations in LRI versus HER, and 20-fold elevations in LRI vs APP. Most transcripts, as demonstrated by CXCR2/IL8Rß, LILRA3, and ALPL, showed roughly similar changes in LRI and APP. Of the 37 transcripts, 10 are relatively selective for APP, 8 are selective for LRI, and 19 behave similarly in both APP and LRI.

### Validation of selected biomarkers using ddPCR in a larger cohort

**Fig. 5:**
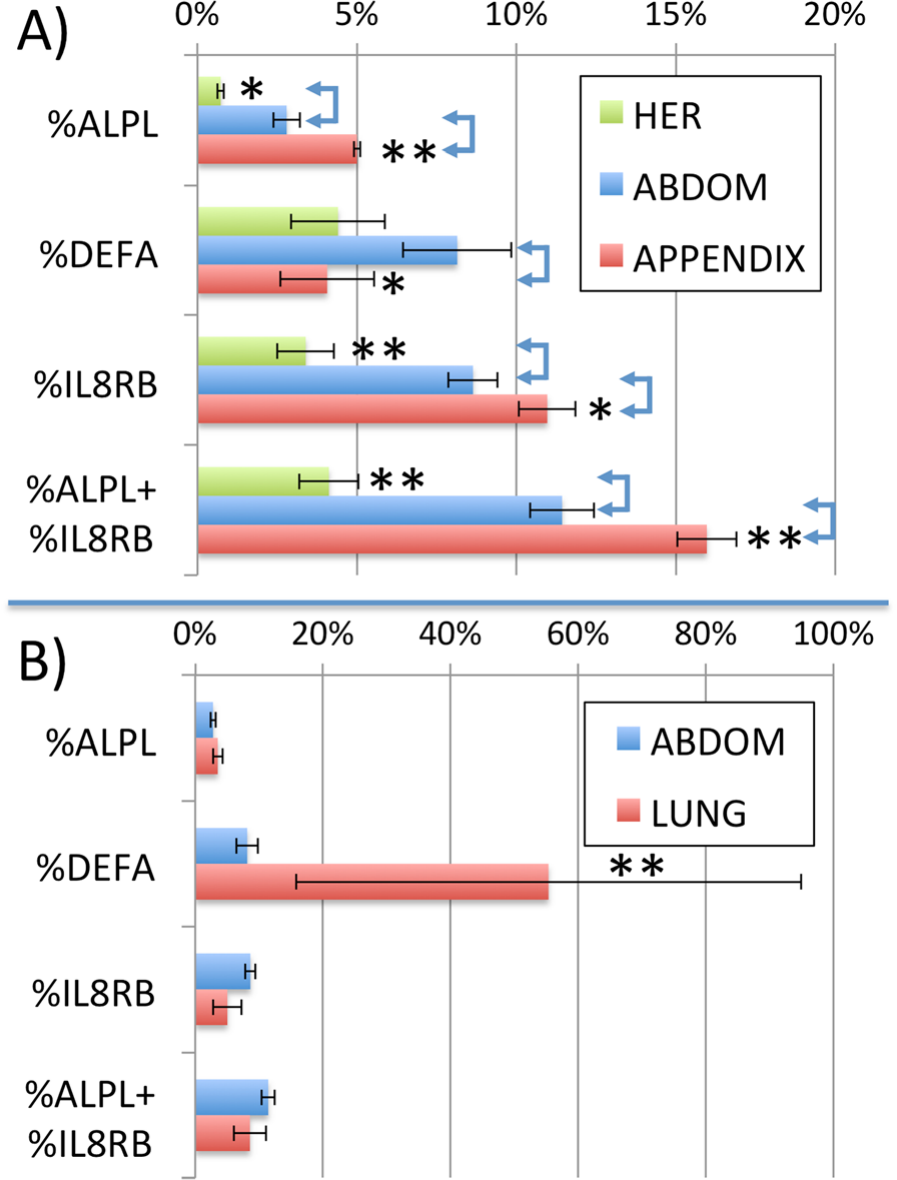
ddRT-PCR validation of selected mRNA biomarkers in an expanded cohort. Patient samples from the discovery and validation sets were expanded to include an additional set of appendicitis and abdominal pain patients drawn from the same cohort. Panel **a** ALPL, DEFA, IL8RB mRNA was quantitated by ddRT-PCR and then expressed as percent of the ACTB value from each subject. Mean and SEM are shown for patients with non-appendicitis abdominal pain (ABDOM, *n* = 30) versus confirmed appendicitis (APPENDIX, *n* = 30). Panel **b** The same biomarkers are shown for ABDOM (*n* = 30) versus suspected lung infections (LUNG, *n* = 5). In both panels, * indicates *p* < 0.05, **indicates *p* < 0.005

To confirm and extend the microarray-based studies, an additional 29 patients (12 APP, 17 ABD) were recruited and combined with all available samples from the prior studies to quantitate the RNA biomarkers using droplet digital PCR (ddPCR) as an independent method. As shown in Fig. 5a, the results confirm the microarray findings that appendicitis patients show significantly elevated circulating levels of mRNA for ALPL and IL8RB, while showing reduced levels of DEFA1 compared to patients with other abdominal conditions (ABD). Abdominal patients showed significantly elevated ALPL, IL8RB, and DEFA1 compared to patients with hernia (HER). Thus, APP patients are characterized as having elevated ALPL and IL8RB, without an increase in DEFA1. In contrast, patients with active lung infections showed strongly activated DEFA1 mRNA levels with only small changes in ALPL and IL8RB (Fig. 5b). The relatively high standard error in the lung group (LRI) is because 2 of the 5 patients had marginal infections, one associated with a primary diagnosis of pulmonary embolism, and the other with a mild respiratory infection, and both showed small increases in DEFA1 levels. A separate ongoing study is examining these markers in pulmonary infections, but the validity of DEFA1 mRNA is supported by recent microarray studies [25].

## Discussion

Currently, there are no approved serum or urine biomarkers for appendicitis. As noted earlier, abdominal pain is one of the most common complaints in the ED, and thus blood biomarkers represent an important unmet need in clinical medicine. In this discovery and validation study, we have identified a small set of RNA transcripts associated with appendicitis. Overall, a prediction model built on these markers was able to differentiate appendicitis from other forms of intra-abdominal pathology, such as diverticulitis and hernias. Appendicitis is thought to be an inflammatory disease, similar to diverticulitis or colitis; however, there was differing activation of certain mRNA biomarkers between these conditions. Furthermore, the 37 DEG markers do not correlate with white blood cell count, per se, but a careful examination of the transcripts suggests that the RNA biomarkers may be measuring the activation state of immune cells, especially neutrophils.

**Fig. 6:**
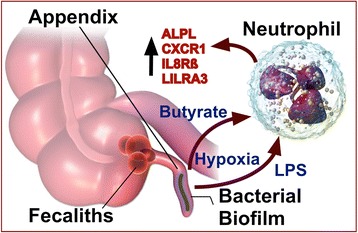
Model of appendicitis biomarker pathophysiology. It is believed that compacted fecal bodies, termed fecaliths, may occlude the outflow tract of the appendix, causing inflammatory conditions that are conducive to infection in the appendix. Microbiome analysis of inflamed appendices typically indicates a predominance of biofilm-forming bacteria, such as Fusobacteria. The biofilm protects the bacteria from antibiotics, and from direct immune attack, but soluble factors produced by the bacteria, such as LPS (endotoxins) and butyrate, or cellular factors such as hypoxia and CXCL8, can diffuse into adjacent lymphatic and circulatory beds to activate neutrophils. The primed neutrophils respond with elevated transcript levels of alkaline phosphatase (ALPL), interleukin-8 receptor beta (CXCR2/IL8Rß) and related biomarkers of local infection. Background images of appendix and neutrophil courtesy of Blausen.com staff, *Wikiversity Journal of Medicine*

The pattern of transcriptome changes in blood may help to refine our understanding of the etiology and progression of acute appendicitis, as shown schematically in Fig. 6. The classic explanation for appendicitis is that a fecalith or lymphoid hyperplasia blocks the outflow of the appendix, resulting in obstruction and ischemia [26]. Outflow obstruction may produce local changes that favor undesirable changes in the appendix microbiome. Several recent studies, including next-generation sequencing (NGS) of the 16S regions of the microbiome, have suggested that relatively selective changes in Fusobacteria species are associated with appendicitis [27–30]. Fusobacteria, a type of gram-negative bacteria, can induce toxicity in adjacent host cells, and colitis-like symptoms in mice, potentially by producing butyric acid (butyrate) [31].

RT-PCR analysis confirms that inflamed appendix tissue has elevated α-defensin and CXCL8 (IL-8) mRNA levels [32]. Most studies have observed higher circulating CXCL8 levels in patients with appendicitis [33–35], and the inflamed appendix is known to locally express elevated CXCL8 [36]. Likewise, *Fusobacterium nucleatum* biofilms stimulate CXCL8 production in human oral epithelium cell lines [37] and *Fusobacterium necrophorum* induces CXCL8 production in cultured mesothelial cells [38]. Neutrophils attracted to sites of mucosal inflammation are a major component of the normal maintenance of barrier functions, and they are responsive to a variety of soluble signals within the intestinal microenvironment, including hypoxia [39].

Thus, the absence of elevated α-defensin transcripts in the presence of elevated levels of mRNA for both CXCL8/IL-8 receptors suggests that circulating immune cells are primed by CXCL8, LPS, butyrate, and local hypoxia known to be produced in the inflamed appendix. However, it is possible that the immune cells are not directly contacting the bacterial infection, which would elevate defensins, as demonstrated clearly in the LRI patients. The increased mRNA levels for ALPL, which is a secondary granule constituent, as opposed to defensins and MPO, which are azurophilic granule components, is consistent with the pre-existing view that secondary granules respond to particulate and soluble neutrophil stimulators, while the azurophilic granule is principally responsive only to phagocytizable particles [40].

In addition to the CXCL8 receptors, several other transcripts appear to be plausible biomarkers of localized inflammation. Notably, ALPL, along with IL8RB/CXCR2, was identified as an expression biomarker of asthma inflammatory subtypes [41]. In addition to these interesting innate immune markers, the results revealed unexpected changes in the ribosomal system. Humans utilize 4 ribosomal RNAs, which are non-coding (5S, 5.8S, 18S, 28S), and ~80 ribosomal proteins to build multimeric translation complexes. Additionally, there are ~2000 ribosomal protein pseudogenes, which are thought to derive from inactivated duplications, but may be processed to varying degrees, and could have regulatory functions [42]. Transcripts for 18S and 28S, both originating from multiple 45S genes, were increased in the appendicitis blood RNA, which could be due to both increased transcription from active rDNA genes [43], as well engagement of previously inactive rDNA transcription units [23]. Conversely, most of the coding transcripts, such as RPLP1 and RPS26, were decreased in the blood of appendicitis patients. Because the specific pattern of ribosomal proteins defines the type of RNAs that are engaged and translated [44], it is possible that the translational machinery is being re-geared to react to pathogens. Unexpectedly, most of the poorly annotated transcripts were mapped to ribosomal protein pseudogenes, suggesting that either the probesets are incorrectly detecting a change in coding ribosomal protein transcripts, or the pseudogenes are somehow regulated in conjunction with the reconfigured translational machinery. Nonetheless, the ribosomal transcript changes reflect a small fraction of the ribosomal machinery, not a sweeping change in ‘housekeeping’ genes. Conceptually, the pattern of chemokine, defensin, stress-related, and ribosomal processing changes is consistent with the immune system being ‘primed’ as the immune cells pass through an inflammatory field created by a localized biofilm infection.

Other transcripts were readily associated with tissue injury or inflammation, but not previously associated with pathogen infection (Table 2). For instance, NINJ1 was identified as a transcript strongly upregulated after peripheral nerve injury [45]. PROK2 is elevated in colitis tissue [46], which, like appendicitis, is an inflammatory condition in the GI tract. Likewise, ALPL has a well-known role in modulating diverse inflammatory conditions not limited to infectious disease [47]. Consistent with the current data, human neutrophils treated *ex vivo* with TNF-α and/or GM-CSF also show induction of transcripts for CXCR1, CXCR2/IL8RB, FCGR3B, NINJ1, and PROK2 [48].

Other investigators have sought to develop protein biomarkers for appendicitis in the blood, such as bilirubin [49], C-reactive protein (CRP) [50], and pro-calcitonin (PCT) [51]. However, recent comparisons of these biomarkers had difficulty improving on a purely clinical prediction model, such as the Alvarado score (ROC = 0.74, vs CRP = 0.61, PCT = 0.69) [52]. Recently, a combination of WBC, CRP, and MRP8/14 (S100A8/S100A9) was shown to be 96 % sensitive, but 43 % specific for acute appendicitis [50]. Likewise, a multivariate model built on plasma protein levels of serum amyloid (SAA), myeloperoxidase (MPO), and MMP9 was less diagnostic than a largely clinical model (ROC = 0.71 vs 0.91 clinical model) [53].

While RNA-based diagnostic tests are currently on the market for breast cancer progression (MammaPrint, OncoType Dx), transplant rejection (AlloMap), and coronary artery disease (CorusCAD), to our knowledge, this is the first report to assess blood RNA as a potential biomarker of appendicitis. Among the strengths of the present approach is that the test and validation sets included controls for surgical, inflammatory, and infectious factors. Further, the RNA profiling was broad and largely unbiased, and detected the same key pathways in the test and validation study. Third, unlike protein biomarkers, which can be difficult to measure due to interference by other high-abundance proteins, mRNA is highly reproducible, very sensitive to perturbation, and can be localized to specific cell types (e.g. neutrophils) for disease monitoring.

In addition to these strengths, the present study has certain limitations. First, the sizes of the cohorts used in the discovery phase were modest. As a consequence, the range of intra-abdominal diseases that we were able to assess was limited. The LRI biomarkers must be assessed cautiously due to the small and heterogeneous group. Second, due to randomness in enrollment, the ethnicity was unbalanced between groups, and this may have introduced a bias into the interpretation. However, analysis of the ddPCR data by race and gender did not identify differences (not shown). Nonetheless, we have successfully utilized a genome-wide RNA transcript profiling to identity potential genomic biomarkers of appendicitis. Overall, the detected biomarkers are consistent with prior published evidence that bacterial biofilms in the appendix may be an important putative pathophysiological mechanism in appendicitis that can be detected by the RNA profile of circulating neutrophils.

## Conclusions

- Transcript profiling of blood identified RNA biomarkers in patients with confirmed appendicitis.
- Secondary granule markers, such as ALPL were increased, while azurophilic granule markers, such as DEFA1, decreased in appendicitis.
- Lung infections increased azurophilic markers more than secondary granule markers.
- Transcript changes associated with acute appendicitis are consistent with immune priming by a bacterial biofilm, probably transmitted by soluble factors such CXCL8, LPS, and local hypoxic conditions that activate neutrophils transiting the inflamed area.

## Additional files

Additional file 1: Table S1. Inclusion and Exclusion Criteria. Describes the inclusion criteria for the patients enrolled in the prospective, observational study of biomarkers for appendicitis. (DOCX 55 kb) Additional file 2: Table S2. Clinical Information on all Patients Analyzed. Describes the age, gender, race, body mass index (BMI), smoking, white blood cell count (WBC), pathology of the appendix, computed tomography (CT) results, and final diagnosis of the patients included in the final analysis. (XLSX 16 kb) Additional file 3: Table S3. Appendix List of Primers. (XLSX 54 kb) Additional file 4: Table S4. Sixteen gene list highly weighted in 37 gene PLS model. Describes the 16 gene transcripts that were the most highly weighted from the larger 37 gene lists. These 16 transcripts were used to build a second PLS prediction model as described. (DOCX 72 kb)
