# Supplementary material for: Acute appendicitis: transcript profiling of blood identifies promising biomarkers and potential underlying processes
Source: BMC Med Genomics. 2016 Jul 15;9:40. doi: 10.1186/s12920-016-0200-y (PMC4946184; doi:10.1186/s12920-016-0200-y)
Supplement: Additional file 1: Table S1. — Inclusion and Exclusion Criteria. Describes the inclusion criteria for the patients enrolled in the prospective, observational study of biomarkers for appendicitis. (DOCX 55 kb) [file 12920_2016_200_MOESM1_ESM.docx]

**Supplementary Table 1: Inclusion and Exclusion Criteria**

| *Inclusion Criteria:* | |
| --- | --- |
|  | |
| (1)  Patients who are being evaluated for acute abdominal pain, including appendicitis or suspected bowel inflammation and/or ischemia. | |
| (2)  Patients undergoing elective non-intestinal surgery, such as hernia repair. | |
| (3)  Patients being treated for pneumonia and/or upper respiratory infection. | |
| (4)  Males and Females of at least 18 years of age able to provide informed consent. | |
|  | |
| *Exclusion Criteria:* | |
|  | |
| (1)  Patients who are moribund or who cannot tolerate a surgical intervention. | |
| (2)  Pregnant patients. | |
| (3)  Patients under the age of 18. | |
