# Supplementary material for: Acute appendicitis: transcript profiling of blood identifies promising biomarkers and potential underlying processes
Source: BMC Med Genomics. 2016 Jul 15;9:40. doi: 10.1186/s12920-016-0200-y (PMC4946184; doi:10.1186/s12920-016-0200-y)
Supplement: Additional file 4: Table S4. — Sixteen gene list highly weighted in 37 gene PLS model. Describes the 16 gene transcripts that were the most highly weighted from the larger 37 gene lists. These 16 transcripts were used to build a second PLS prediction model as described. (DOCX 72 kb) [file 12920_2016_200_MOESM4_ESM.docx]

**Supplementary Table 4. Sixteen gene list highly weighted in 37 gene PLS model.**

| **PROBE_ID** | **SYMBOL** | **FC (abs)** | **Change** | **ABDOM**  **level** | **APP**  **level** |
| --- | --- | --- | --- | --- | --- |
| ILMN_1701603 | ALPL | 2.84 | up | 3.63 | 5.14 |
| ILMN_1761566 | C5orf32 | 2.15 | up | 1.10 | 2.21 |
| ILMN_1697499 | HLA-DRB5 | 3.16 | up | -0.11 | 1.55 |
| ILMN_1680397 | CXCR2 | 2.59 | up | 2.80 | 4.17 |
| ILMN_1661631 | LILRA3 | 2.36 | up | 2.38 | 3.62 |
| ILMN_3243593 | LOC100008588 | 2.69 | up | 1.04 | 2.47 |
| ILMN_1733559 | LOC100008589 | 2.05 | up | 1.20 | 2.23 |
| ILMN_3249578 | LOC100132394 | 2.81 | up | 1.69 | 3.18 |
| ILMN_3246805 | LOC100134364 | 2.04 | up | 1.17 | 2.21 |
| ILMN_3293367 | LOC391370 | 2.01 | down | 2.11 | 1.10 |
| ILMN_3209193 | LOC644191 | 2.00 | down | 2.23 | 1.23 |
| ILMN_2155719 | NBPF10 | 2.01 | down | 3.56 | 2.55 |
| ILMN_1815086 | NINJ1 | 2.10 | up | 2.61 | 3.68 |
| ILMN_1775257 | PROK2 | 2.49 | up | 3.30 | 4.62 |
| ILMN_1755115 | RPL23 | 2.14 | down | 1.49 | 0.39 |
| ILMN_2336781 | SOD2 | 2.02 | up | 3.43 | 4.44 |
